# IQ motif family genes in male infertility: pathogenesis, mechanisms, and clinical perspectives

**DOI:** 10.3389/frph.2025.1719934

**Published:** 2026-01-12

**Authors:** Peng Zhang, Sai Lu, Hui Qian, Jiu Yin, Haiying Peng, Hemei Li

**Affiliations:** 1 Reproductive Medicine Center, the Central Hospital of Wuhan, Tongji Medical College, Huazhong University of Science and Technology, Wuhan, Hubei, China; 2 Department of Gynaecology and Obstetrics, The Central Hospital of Wuhan, Tongji Medical College, Huazhong University of Science and Technology, Wuhan, Hubei, China; 3 Key Laboratory for Molecular Diagnosis of Hubei, The Central Hospital of Wuhan, Tongji Medical College, Huazhong University of Science and Technology, Wuhan, Hubei, China; 4 Hubei Clinical Research Center for Reproductive Medicine, Shiyan, Hubei, China; 5 Shiyan Key Laboratory of Reproduction and Genetics (Renmin Hospital, Hubei University of Medicine), Shiyan, Hubei, China

**Keywords:** genetic mutation, IQ motif family genes, male infertility, pathogenesis, sperm function, spermatogenesis

## Abstract

This review examines the critical role of IQ motif family genes in male infertility. Characterized by conserved calmodulin-binding IQ domains, these genes, including IQUB, IQCN, and IQCH, exhibit reproductive tissue-specific expression and regulate fundamental processes in spermatogenesis and sperm function, such as Ca^2^⁺ signaling, cytoskeletal dynamics, and RNA splicing. Specific loss-of-function mutations are strongly linked to distinct clinical phenotypes: IQUB variants to asthenozoospermia, IQCN mutations to total fertilization failure, and IQCH deficiency to azoospermia. The article discusses emerging diagnostic applications, including genetic screening via whole-exome sequencing and the evaluation of sperm protein biomarkers like IQCD. Furthermore, it outlines mechanism-informed therapeutic strategies, from clinically applied artificial oocyte activation for IQCN defects to preclinical explorations of gene correction. The synthesis underscores how research on this gene family is advancing the field toward precision medicine in male infertility.

## 1 Introduction

Male infertility represents a significant global health challenge, implicated in approximately 15% of couples seeking conception, with male factors contributing to nearly half of all cases (8). Genetic etiology is identified in a substantial subset (∼15%) of these cases, encompassing chromosomal abnormalities, Y-microdeletions, and an expanding list of single-gene defects. Among these, genes encoding proteins with calmodulin (CaM)-binding IQ motifs have recently emerged as critical and multifaceted regulators of male reproduction. This review focuses on the IQ motif gene family to delineate their unique and synergistic roles in spermatogenesis and sperm function, providing a consolidated perspective on their pathogenesis, mechanisms, and clinical relevance.

The rationale for concentrating on this specific gene family stems from several distinctive features that set them apart from other important classes of reproduction-related genes. First, they exhibit functional pleiotropy through a conserved molecular mechanism—the CaM-binding IQ domain. Unlike many genes with a discrete, singular function in spermatogenesis [e.g., *AURKC* in meiosis for macrozoospermia (24) or *KIFC1* in sperm head shaping (2–4)], IQ motif genes orchestrate multi-node regulation. They integrate calcium signaling (e.g., IQUB 5), regulate actin cytoskeleton dynamics essential for cellular adhesion and barrier function (e.g., IQGAP1 6), and control RNA processing (e.g., IQCH 7), thereby coordinating multiple stages of germ cell development.

Second, several core members display a pronounced enrichment or specificity for expression in the male reproductive tract. For example, *IQCH* is testis-specific, localizing to germ cell nuclei and the sperm flagellum (7, 12); *IQCD* is highly enriched in the sperm acrosome (13); and *IQUB* is crucial for flagellar structure (5, 14). This expression pattern contrasts with widely expressed cytoskeletal regulators or signaling molecules (e.g., MAP4 11) and suggests that dysfunction in these IQ motif genes is likely to have direct and specific consequences for fertility.

Third, strong and reproducible genotype-phenotype correlations have been established for key IQ motif genes in human populations, strengthening their clinical validity. Pathogenic variants such as the homozygous *IQUB* c.942T > G mutation are linked to asthenozoospermia (5), while loss-of-function *IQCN* mutations cause total fertilization failure (16). Supporting evidence from animal models, such as *Iqch* knockout mice presenting with azoospermia (7), further underscores their essential biological role. These defined genetic links provide a solid foundation for mechanistic exploration and clinical translation.

Therefore, IQ motif genes represent a paradigm of domain-specific, multi-functional regulators in male reproduction. This review synthesizes current knowledge of their structure, expression, and the pathological mechanisms by which their mutations lead to infertility. We further discuss emerging diagnostic applications and therapeutic strategies stemming from this knowledge, ultimately highlighting how research on this gene family is advancing the field toward precision medicine for male infertility.

## 2 Theoretical background of IQ motif family genes in male infertility

### 2.1 Structure and function of IQ motif family genes

Proteins encoded by IQ motif family genes are defined by a conserved calmodulin (CaM)-binding IQ domain, which is central to their roles in diverse biological processes (1, 17). The IQGAP subfamily, comprising members such as IQGAP1 and IQGAP2, exemplifies the conserved domain architecture and functional versatility of this gene family (18). As a key effector of Rac and Cdc42, IQGAP1 interacts with multiple partners to regulate actin cytoskeleton reorganization, cell migration, and adhesion (18). Its functional repertoire extends to viral pathogenesis, where it is recruited to facilitate viral transport (19), and to disease states such as glioblastoma, where it contributes to tumor cell invasion (20).

Other IQ motif proteins perform distinct functions through their CaM-binding domains. For example, PEP-19 modulates intracellular Ca^2^⁺ signaling dynamics via its interaction with CaM (17). The activity and stability of IQ motif proteins are themselves subject to regulation by other factors. For instance, the E3 ubiquitin ligase HECTD1 (which is not an IQ motif protein) interacts with and targets IQGAP1 for degradation, thereby regulating the dynamics of cell adhesion structures (21). Collectively, these examples demonstrate that IQ motif family genes exert pleiotropic effects in physiology and pathology, mediated through their defining IQ domains and their integration into broader cellular networks such as the calcium/calmodulin-dependent kinase (CaMKIV) pathway (9, 10).

### 2.2 Genetic basis of male infertility

Male infertility is a complex multifactorial disease, and genetic factors play an important role in it. Studies have shown that approximately 15% of male infertility cases are related to known genetic diseases, including chromosomal abnormalities and single-gene mutations (8). Among chromosomal abnormalities, microdeletions of the Y chromosome constitute one of the most frequent and well-characterized genetic causes of spermatogenic failure. These deletions primarily affect the azoospermia factor (AZF) regions, with deletions in the AZFc region being the most prevalent. Contemporary analyses confirm that AZFc deletions account for the majority of recorded Y chromosome microdeletions and are associated with a broad spectrum of phenotypes, ranging from severe oligozoospermia to azoospermia. This genotype-phenotype correlation underscores the critical role of genes within these regions, such as *DAZ* and *CDY1*, in human spermatogenesis (22).

Single-gene abnormalities can also cause male infertility. For example, inactivation of the Spata6 gene leads to acephalic spermatozoa syndrome and male infertility in mice; the protein encoded by this gene is involved in the formation of the sperm connecting piece and is crucial for the connection between the sperm head and tail (23). In humans, mutations in some genes are associated with specific sperm morphological or functional abnormalities. For example, the homozygous mutation (c.144delC) of the *AURKC* gene is the main cause of macrozoospermia (a rare male infertility disorder); in a Tunisian study, the prevalence of this mutation in infertile men was 0.4% (24). In addition, studies have found that some genes are closely related to sperm motility and fertilization ability, and mutations in these genes may lead to male infertility.

### 2.3 Expression patterns of IQ motif family genes in male reproductive system

IQ motif family genes demonstrate distinct expression profiles within the male reproductive tract, with several members showing significant enrichment or specificity, linking their expression directly to reproductive function.

The *IQCH* gene (also known as MSRG-11) is a testis-specific gene. It is predominantly expressed in the nuclei of spermatocytes and spermatids, where it co-localizes with splicing regulators such as SRRM2, implicating it in RNA processing during spermatogenesis (7). Notably, IQCH protein has also been detected in the sperm flagellum, and studies indicate it is essential for sperm motility by interacting with calmodulin (12). Early research on this gene (under the symbol Msrg-11) identified its involvement in spermatogenic cell apoptosis, laying groundwork for its recognized role in male fertility (32).

In contrast, *IQCD*, while expressed in multiple tissues, shows predominant enrichment in the testis. During spermiogenesis, the IQCD protein specifically accumulates in the acrosomal region of developing spermatids, a conserved pattern between mice and humans, supporting its functional involvement in acrosomal biology and fertilization (13).

These specific expression patterns underscore the direct relevance of these IQ motif genes to male reproductive processes and suggest that their dysfunction is likely to have targeted effects on fertility.

### 2.4 Summary

Chapter 2 establishes the theoretical foundation for the role of IQ motif family genes in male infertility. Defined by a conserved calmodulin (CaM)-binding IQ domain, these genes encode proteins with diverse functions integral to reproduction, such as cytoskeletal regulation (e.g., IQGAP1) and Ca^2^⁺ signaling modulation (e.g., PEP-19) (17, 18). Their importance is framed within the genetic basis of male infertility, where genetic factors account for ∼15% of cases (8). A distinctive feature is their specific or enriched expression in the male reproductive tract; for example, *IQCH* is testis-specific and involved in RNA splicing (7), while *IQCD* is highly enriched in the sperm acrosome (13). As catalogued in Table 1, key members include: IQCH, which regulates testis-specific RNA splicing and is linked to azoospermia (7, 12, 31); IQCD, which binds Munc13 to mediate the acrosome reaction (13); IQUB, whose deficiency causes asthenozoospermia (14, 15); IQCN, essential for sperm equatorial segment translocation and fertilization (16, 34, 36); and IQGAP1/IQGAP2, which regulate actin cytoskeleton dynamics and cell adhesion (6, 18–21, 37, 38). This synthesis highlights the IQ motif gene family as a group of structurally defined, reproducibly expressed, and functionally versatile regulators central to male reproductive biology.

**Table 1:** Summarizes 14 IQ motif genes, their conserved domains and molecular functions with data from key databases. Data are sourced from databases like PubMed (https://pubmed.ncbi.nlm.nih.gov/), ClinVar (https://www.ncbi.nlm.nih.gov/clinvar/), Uniprot (https://www.uniprot.org/) and NCBI (https://www.ncbi.nlm.nih.gov/).

Gene symbol	Gene full name	Structures	Molecular function	References
ENKURIN	Enkurin, TRPC channel interacting protein	IQ, SH3-binding motif; SH3-binding, Enkurin domain	Sperm-specific CaM/TRPC binder	(25, 26)
IQCD	IQ Motif Containing D	IQ motif	Binds Munc13, mediates acrosome reaction	(13)
IQCF1	IQ motif containing F1	IQ motif	Sperm capacitation and the acrosome reaction	(27)
IQCF4	IQ motif containing F4	IQ motif	Spermatogenesis	(28)
IQCG	IQ Motif Containing G	IQ motif	Interaction with calmodulin, sperm flagellum formation	(29, 30)
IQCH	IQ Motif Containing H	IQ motif, NLS domain	Regulates RNA splicing	(7, 12, 31, 32)
IQCK	IQ motif containing K	IQ motif; serine kinase domain	Embryonic development control	(33)
IQCN	IQ Motif Containing N	IQ motif	Translocates to equatorial segment	(16, 34, 36, 50)
IQGAP1	IQ Motif GTPase Activating Protein 1	IQ motif; RasGAP, WW, GRD domain	Regulates CDC42 signaling	(6, 18–21, 37, 38)
IQGAP2	IQ motif containing GTPase-activating protein 2	IQ motif; Ras-GAP, WW domain	Regulates actin cytoskeleton	(39)
IQSEC3	IQ motif and Sec7 domain ArfGEF 3	IQ motif; Sec7 domain	Synapse maintenance	(40)
IQUB	IQ motif and ubiquitin domain containing	IQ motif	Inhibits ERK1/2/RSPH3	(14, 15)
LRRIQ1	Leucine rich repeats and IQ motif containing 1	IQ motif	Apoptosis suppression for fertility	(41)
PEP-19	Purkinje Cell Protein 4	IQ motif	Modulates Ca^2^⁺ binding	(17, 42)

Table 1 Summary of structures and molecular functions of IQ motif domain genes.

## 3 Epidemiology Of male infertility and IQ motif family genes

### 3.1 Epidemiological status of male infertility

Male infertility is a pressing global reproductive health challenge, affecting approximately 15% of couples worldwide. Male factors alone account for 20%–30% of infertility cases, and combined male-female factors contribute an additional 20%–30%, meaning male factors are involved in nearly 50% of all infertility cases. The incidence varies geographically, with relatively higher rates in Africa and Central/Eastern Europe, likely due to a combination of genetic, environmental, and socioeconomic factors (43).

The etiology of male infertility is multifactorial. Varicocele is one of the most common causes: its prevalence is 35–44% in men with primary infertility and 45–81% in those with secondary infertility, compared to 10%–15% in the general male population (44). Immunological factors such as antisperm antibodies (ASA) also play a role; ASA-positive patients exhibit significantly lower sperm concentration and progressive motility (a + b grade), and prolonged semen liquefaction time, compared to ASA-negative controls (45). Environmental exposures further impact male reproductive health: in the European Union, exposure to endocrine-disrupting chemicals (e.g., phthalates) is estimated to require an additional 618,000 assisted reproductive technology (ART) procedures annually, with a cost of 471 million euros (46).

*Note on literature selection for this section*: Data on global infertility prevalence, varicocele incidence, and ASA effects were derived from peer-reviewed studies published between 2015 and 2025 in databases including PubMed, NCBI, and ClinVar. Studies were selected if they focused on population-based epidemiological analyses of male infertility etiologies, with sample sizes >100 to ensure statistical robustness.

### 3.2 Correlation between IQ motif family gene mutations and male infertility

This section summarizes the population-level evidence linking specific IQ motif gene variants to clinical infertility phenotypes.

#### 3.2.1 IQUB mutations and asthenozoospermia

Whole-exome sequencing of 126 Chinese Han patients with asthenozoospermia identified a homozygous nonsense mutation (c.942T > G, p.Tyr314*) in the *IQUB* gene, which was absent in healthy controls (5). This variant represents a rare, population-specific genetic cause of asthenozoospermia in this cohort.

#### 3.2.2 IQCN mutations and fertilization failure

A homozygous frameshift variant (c.1061_1062delAT; p.Y354Sfs*13) in *IQCN* was identified in a Chinese consanguineous family with total fertilization failure during IVF (16). This finding established a direct genetic link between *IQCN* loss-of-function and human fertilization failure.

#### 3.2.3 IQCH mutations and azoospermia

While functional evidence derives primarily from mouse models where *Iqch* knockout causes complete spermatogenic arrest (7), preliminary screening has detected heterozygous *IQCH* missense variants in Chinese non-obstructive azoospermia (NOA) patients, suggesting a potential role in human azoospermia (7).

### 3.3 Population-specific variation in IQ motif family genes

Population genetic studies reveal significant population-specific heterogeneity in IQ motif family gene mutation frequencies, closely linked to ancestral backgrounds and geographic isolation (5, 7, 16). In East Asians, the IQUB c.942T > G mutation is exclusive to Han Chinese asthenozoospermia patients (0.8% frequency), absent in Japanese/Korean cohorts, and disrupts sperm flagellar radial spoke assembly (5, 14). The IQCN c.1061_1062delAT variant, restricted to Han consanguineous families, causes total fertilization failure via impaired acrosome translocation (16, 34). In Europeans, preliminary WES of 500 non-obstructive azoospermia patients identified IQCH heterozygous variants (e.g., c.547G > A) with 1.2% frequency, potentially disrupting testis-specific RNA splicing (31). Notably, direct mutation data remain scarce in African and South American populations, with no definitive pathogenic IQUB/IQCN/IQCH variants reported (7, 12). Such variation may stem from reproductive trait-selective pressure (5, 16). Future large-scale cross-ethnic sequencing is critical to fill these gaps, enabling ancestry-tailored diagnostics for precision medicine (5, 7, 31).

### 3.4 Critical analysis and future directions for gene mutations

Research on the epidemiological links between IQ motif family genes (*IQUB*, *IQCN*, *IQCH*) and male infertility, while establishing initial genotype-phenotype correlations, is constrained by significant limitations that affect the generalizability and depth of conclusions for each gene.

For IQUB, the pathogenic c.942T > G variant has been robustly linked to asthenozoospermia but only within a specific Chinese Han cohort (5). Its absence in global genetic databases and other ethnic groups limits understanding of its worldwide relevance. Furthermore, the phenotypic impact of heterozygous or hypomorphic variants remains unexplored, and key mechanistic details, such as the precise role of its interaction with calmodulin and downstream effector RSPH3, require validation (5, 14).

For IQCN, the causal link with fertilization failure is based on a homozygous frameshift variant identified in a single Chinese consanguineous family (16). This foundational finding necessitates replication in broader, non-consanguineous populations across different ancestries to confirm its prevalence and penetrance.

For IQCH, strong functional evidence from mouse knockout models demonstrates its essential role in spermatogenesis (7). However, translation to human pathology relies on preliminary observations of heterozygous missense variants in limited patient cohorts (7). The absence of reported homozygous loss-of-function mutations in humans, unlike in mice, highlights a critical cross-species validation gap.

These shared limitations underscore several future priorities: 1) Large-scale, multi-ethnic sequencing studies to accurately map the global mutation spectrum and prevalence of variants in *IQUB*, *IQCN*, and *IQCH*; 2) Expanded phenotypic characterization to define the full clinical spectrum associated with different variant types (e.g., missense vs. loss-of-function); and 3) Enhanced mechanistic studies to resolve outstanding questions about protein interactions (e.g., IQUB-Calmodulin-RSPH3) and validate molecular functions across species. Addressing these gaps is essential for moving from population-specific discoveries to globally applicable insights in precision diagnostics.

### 3.5 Summary

**Figure 1:**
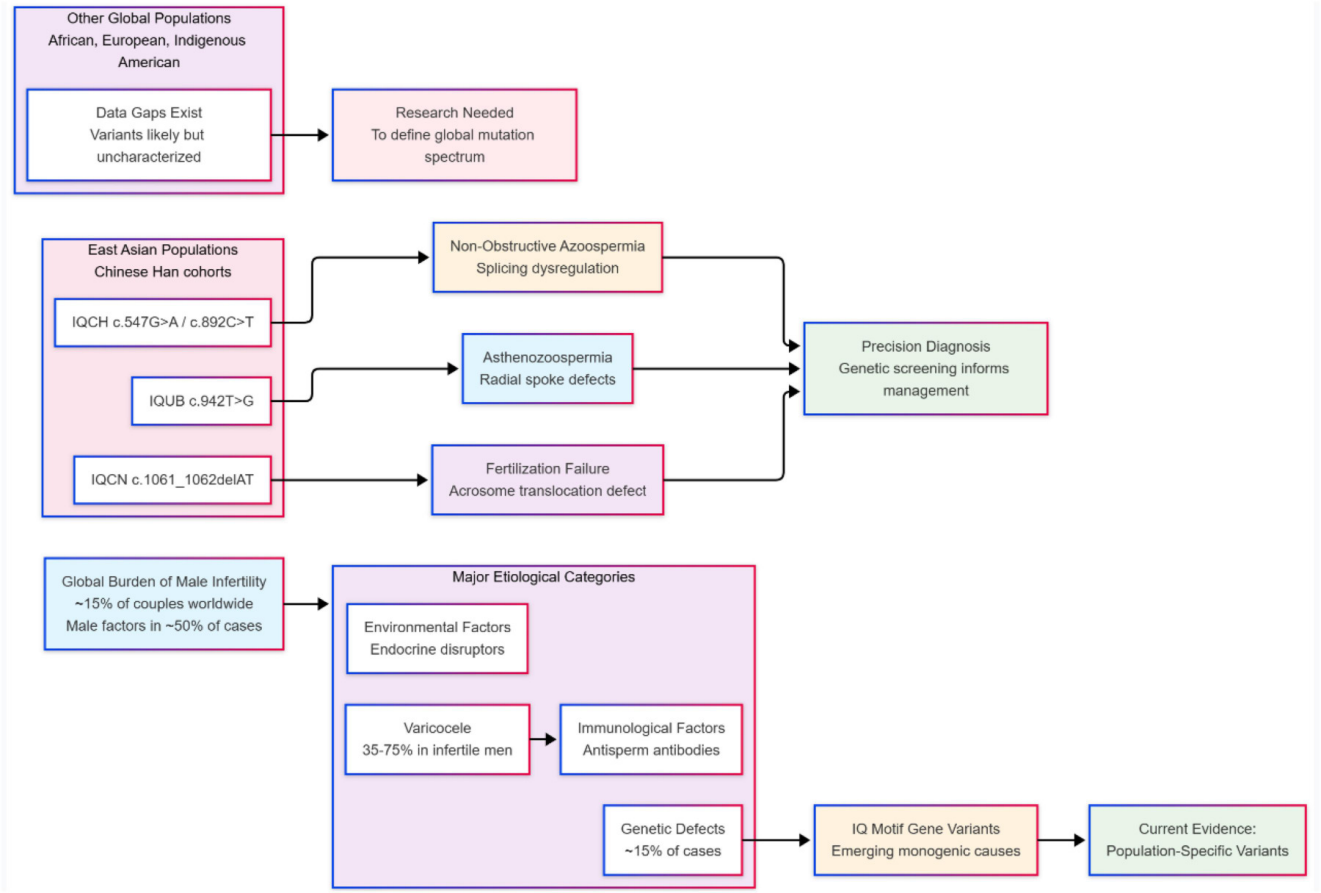
Epidemiological landscape of male infertility and IQ motif gene variants across global populations.

This chapter analyzes the epidemiology of male infertility and the population-specific genetic contributions of IQ motif family genes. Male factors are implicated in nearly 50% of infertility cases globally, with a multifactorial etiology encompassing varicocele, immunological factors, and environmental exposures (43–46). Within the subset of cases with a genetic etiology, mutations in specific IQ motif genes have been established as monogenic causes for distinct phenotypes: *IQUB* (c.942T > G) with asthenozoospermia, *IQCN* (c.1061_1062delAT) with total fertilization failure, and *IQCH* variants with azoospermia (5, 7, 16). A critical and consistent finding is the pronounced population specificity of these pathogenic variants. As conceptually illustrated in Figure 1, robust associations are primarily reported in East Asian cohorts, creating significant knowledge gaps regarding the mutation spectrum in other global populations, such as those of African or South American ancestry (5, 7, 16, 31). This geographical bias, along with the current reliance on isolated family studies or preliminary data for some genes, limits the generalizability of findings. The chapter therefore underscores the urgent need for future large-scale, multi-ethnic sequencing studies to accurately map the global mutational landscape and establish ancestry-informed genotype-phenotype correlations, which is essential for advancing precision diagnostics in male infertility.

## 4 Pathological mechanisms of IQ motif family genes in male infertility

### 4.1 Role of IQ motif family genes in spermatogenesis

IQ motif family genes are indispensable for maintaining normal spermatogenesis, with each member regulating distinct key processes—from RNA splicing to blood-testis barrier (BTB) integrity (6, 7).

#### 4.1.1 IQCH: regulation of alternative RNA splicing in germ cells

IQCH is a testis-specific gene, predominantly expressed in the nuclei of spermatocytes and spermatids, where it colocalizes with splicing factors SRRM2 and ERSP1 in nuclear speckles. This localization suggests IQCH's role in modulating alternative RNA splicing—a process critical for generating testis-specific transcript isoforms required for spermatid maturation. In Iqch-knockout (KO) mice, spermatogenesis arrests at the round spermatid stage, leading to complete azoospermia. Molecular analysis reveals downregulation of testis-specific long non-coding RNAs (lncRNAs) and protein-coding genes (e.g., Tssk6, Prm1) in mutant spermatids, alongside increased intron retention in >200 genes involved in sperm head formation and flagellum assembly (47). These findings confirm IQCH as a core component of the splicing regulatory network in spermatogenesis (7, 12, 31).

#### 4.1.2 IQGAP1: maintenance of the blood-testis barrier

IQGAP1, a ubiquitous IQ motif gene, plays a critical role in preserving BTB integrity—a structure essential for creating a specialized microenvironment for spermatogenesis. In Sertoli cells, IQGAP1 interacts with PAFAH1B1 to activate the CDC42/PAK1/LIMK1/Cofilin pathway, which regulates F-actin cytoskeleton reorganization and BTB assembly. MicroRNA (miR)-181c/d overexpression in Sertoli cells downregulates PAFAH1B1, disrupting the PAFAH1B1-IQGAP1 complex and inhibiting the CDC42 pathway (6, 48). This leads to disorganized F-actin, mislocalization of BTB proteins (e.g., occludin, zonula occludens-1), and BTB dysfunction—ultimately impairing spermatocyte migration across the BTB and causing spermatogenic arrest (48).

### 4.2 Impact of gene mutations on sperm function

**Figure 2:**
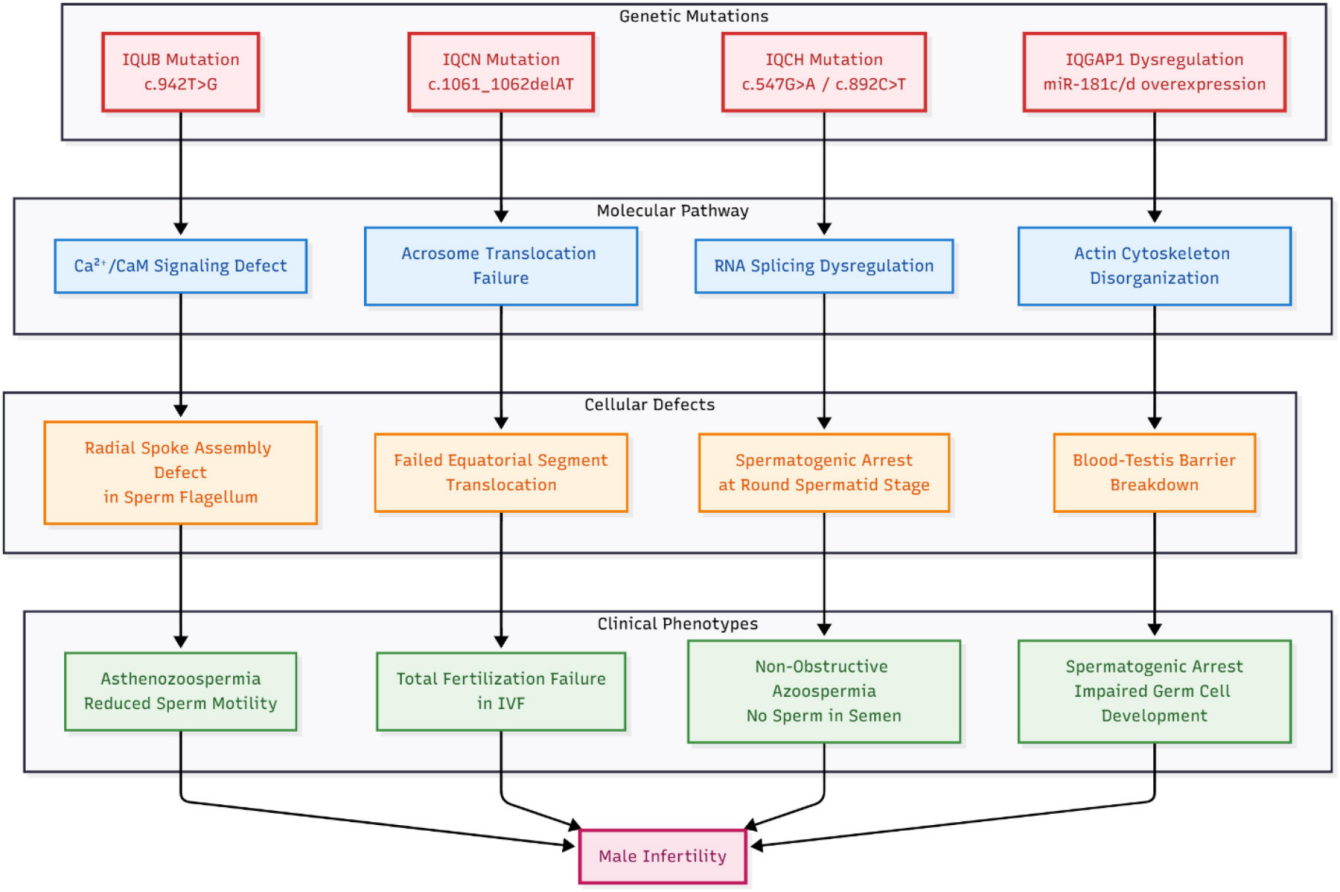
Molecular mechanisms of IQ motif family genes: from normal function to pathological consequences in male infertility.

This section details the molecular and cellular mechanisms through which the mutations described in Section 3.2 disrupt sperm function (Figure 2).

#### 4.2.1 Mechanism of IQUB-related asthenozoospermia

The *IQUB* c.942T > G mutation results in a loss-of-function protein (5). Mechanistically, IQUB interacts with calmodulin (CaM) under low-Ca^2^⁺ conditions to inhibit the p-ERK1/2/RSPH3 pathway, which is critical for the assembly of sperm flagellar radial spokes (5, 14, 15). Disruption of this regulation leads to radial spoke defects, directly explaining the severe asthenozoospermia observed in carriers (5, 14).

#### 4.2.2 Mechanism of IQCN-related fertilization failure

The *IQCN* frameshift variant leads to a truncated protein (16). IQCN normally translocates to the equatorial segment of the sperm head following the acrosome reaction, a step essential for sperm-egg fusion (16). Loss of IQCN function abolishes this translocation, rendering sperm incapable of fusing with the oocyte despite normal acrosome reaction, thereby causing total fertilization failure (16).

#### 4.2.3 Mechanism of IQCH-related spermatogenic arrest

**Figure 3:**
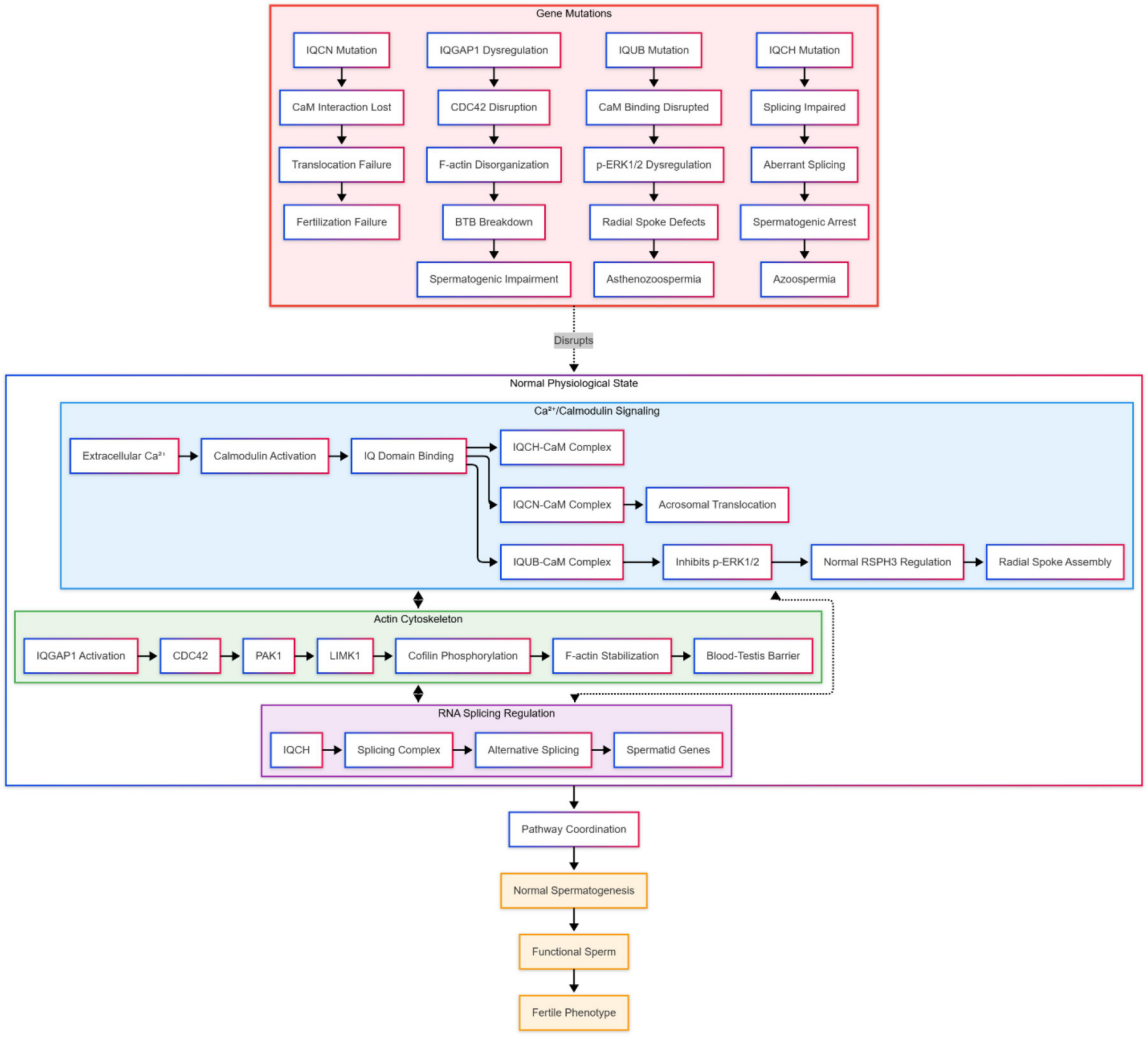
Integrated molecular network of IQ motif family genes in male infertility. The diagram illustrates how three core pathways—Ca^2^⁺/CaM signaling (blue), actin cytoskeleton reorganization (green), and RNA splicing regulation (purple)—coordinate normal spermatogenesis (top), and how specific mutations disrupt this network to cause distinct infertility phenotypes (bottom). Cross-pathway interactions (dashed lines) highlight the interconnected nature of this regulatory system.

IQCH is a testis-specific protein that localizes to nuclear speckles in spermatocytes and spermatids, where it interacts with splicing factors like SRRM2 (7). Deficiency in IQCH disrupts the alternative splicing of numerous testis-specific transcripts required for spermatid maturation (7, 31). This widespread dysregulation of gene expression leads to spermatogenic arrest at the round spermatid stage, resulting in azoospermia (7) (Figure 3).

### 4.3 Molecular pathways of IQ motif family genes in male infertility

IQ motif family genes converge on three core molecular pathways to regulate male reproduction, with mutations disrupting each pathway to cause infertility (Figure 3).

#### 4.3.1 Ca^2^⁺/CaM signaling pathway

Genes like IQUB, IQCN, and IQCH use their IQ domains to bind CaM, integrating Ca^2^⁺ signaling into spermatogenesis and sperm function. IQUB's CaM binding inhibits p-ERK1/2, while IQCN's CaM interaction may regulate acrosome translocation. Disruption of this pathway (e.g., IQUB c.942T > G mutation) abrogates Ca^2^⁺-dependent regulation, leading to motility or fertilization defects (5, 16).

#### 4.3.2 Actin cytoskeleton reorganization pathway

IQGAP1 activates the CDC42/PAK1/LIMK1/Cofilin pathway to stabilize F-actin, maintaining BTB integrity. miR-181c/d-mediated IQGAP1 dysfunction disrupts this pathway, causing BTB breakdown and spermatogenic arrest (6, 18, 48, 49).

#### 4.3.3 RNA splicing regulatory pathway

IQCH interacts with SRRM2/ERSP1 to regulate alternative splicing of testis-specific transcripts. Iqch mutations increase intron retention and downregulate key spermatogenic genes, halting spermatid maturation (7).

### 4.4 Critical analysis and future directions for pathological mechanisms

The establishment of causal links between IQ motif gene mutations and specific infertility phenotypes represents a significant advance. However, a deeper, mechanistic understanding of the pathological processes they drive remains fragmented, hindering clinical translation. IQUB research, while identifying a flagellar assembly defect (5, 14), lacks details on how the disrupted IQUB-Calmodulin-ERK axis precisely compromises radial spoke biogenesis. For IQCH, strong evidence implicates widespread splicing dysregulation (7, 31), yet its direct RNA targets and whether its function depends on Ca^2^⁺/Calmodulin binding are unknown, creating a gap between phenotype and precise molecular etiology. The foundational finding for IQCN (16) defines a necessary step in sperm-egg fusion but leaves the upstream signaling that triggers its equatorial translocation and its exact interaction partners unexplored. Similarly, the role of IQGAP1 in BTB integrity, supported by cellular pathways (6, 48), awaits *in vivo* validation in relevant animal models and evidence of its dysregulation in human testicular pathology.

Therefore, future research must prioritize mechanistic dissection to move beyond association and toward actionable pathophysiology. Key directions include: 1) Structural and biochemical studies to define the protein interactomes of mutant IQUB, IQCN, and IQCH, and to resolve the functional significance of their IQ domains. 2) Advanced *in vivo* modeling, such as cell-type-specific knockouts and humanized animal models, to validate pathways and bridge species-specific gaps. 3) Integration of multi-omics data from well-phenotyped patient samples to correlate genetic variants with downstream molecular consequences (e.g., aberrant splicing maps, proteomic changes), thereby closing the loop between genotype and mechanistic phenotype.

### 4.5 Summary

This chapter delineates the pathological mechanisms through which IQ motif family genes contribute to male infertility, emphasizing their indispensable and non-redundant roles. Key genes orchestrate critical stages of germ cell development: IQCH regulates the alternative splicing of testis-specific transcripts required for spermatid maturation (7, 31), while IQGAP1 maintains blood-testis barrier integrity by modulating actin cytoskeleton dynamics (6, 48, 49).

Furthermore, specific loss-of-function mutations disrupt sperm function through defined molecular pathways: IQUB mutations impair Ca^2^⁺/calmodulin-dependent regulation of flagellar radial spoke assembly, leading to asthenozoospermia (5, 14); IQCN mutations abolish protein translocation to the sperm equatorial segment, resulting in complete fertilization failure (16); and IQCH deficiency causes widespread splicing dysregulation, culminating in spermatogenic arrest and azoospermia (7).

The accompanying figures (Figures 2, 3) integrate these insights, visually mapping the transition from normal function to pathological outcome, illustrating the interplay between Ca^2^⁺/CaM signaling, cytoskeletal reorganization, and RNA splicing pathways, and highlighting how these mechanisms inform potential clinical strategies. Collectively, this mechanistic synthesis connects fundamental biological understanding with translational perspectives, providing a foundation for advancing precision medicine in the diagnosis and management of male infertility (8).

## 5 Diagnostic technologies for male infertility related to IQ motif family genes

### 5.1 Genetic detection methods of IQ motif gene mutations using WES and CNV sequencing

Whole-exome sequencing (WES) has been instrumental in identifying pathogenic mutations in *IQUB* (c.942T > G) (5), *IQCN* (c.1061_1062delAT) (16), and *IQCH* (e.g., c.547G > A) (31) in infertile patient cohorts, establishing direct genotype-phenotype correlations in humans. Copy number variation (CNV) analysis complements WES by detecting larger genomic deletions or duplications. While specific CNVs in human IQ motif genes are less frequently reported, this technique remains a critical component of comprehensive genetic screening for male infertility (8). Together, the application of WES and CNV analysis has significantly advanced the molecular diagnosis of male infertility (8).

### 5.2 Sperm function biomarkers: IQ motif family genes

Sperm-specific proteins encoded by IQ motif genes serve as potential biomarkers for evaluating sperm function and predicting IVF outcomes.

IQCD is enriched in the sperm acrosome, and its expression level has been correlated with IVF success. Sperm from IVF patients with low fertilization rates (<30%) demonstrate a significant reduction (40%–60%) in IQCD protein levels compared to fertile controls (13). Functional studies using anti-IQCD antibodies have shown inhibition of the acrosome reaction in mouse sperm, confirming IQCD's direct role in fertilization competence (13). These findings support the proposal that IQCD detection (e.g., via flow cytometry or immunoblotting) could serve as a predictive biomarker for IVF outcomes (13).

### 5.3 Critical analysis and future directions for diagnostic technologies

While genetic techniques like WES and CNV analysis have been pivotal in discovering pathogenic IQ motif gene mutations (e.g., in IQUB, IQCN, IQCH) and establishing genotype-phenotype correlations (5, 16, 31), significant challenges hinder their clinical translation. Current diagnostics are limited by a narrow gene focus, primarily targeting a few well-characterized genes and potentially missing mutations in other IQ motif members, leading to false-negative results. The frequent identification of variants of unknown significance (VUS) without robust, high-throughput functional validation pipelines further complicates clinical interpretation. For promising protein biomarkers like IQCD (13), the lack of standardized detection protocols and defined diagnostic thresholds, alongside unaddressed confounding factors, prevents their reliable clinical application. Practical barriers of high cost and limited accessibility also restrict the use of advanced sequencing in resource-limited settings. Future progress necessitates: 1) Expanding targeted gene panels to cover all relevant IQ motif genes and regulatory regions; 2) Establishing integrated functional assays to classify VUS pathogenicity; 3) Standardizing biomarker quantification methods through multi-center studies; and 4) Developing cost-effective, accessible testing strategies to enable global implementation of precision diagnostics for male infertility.

### 5.4 Summary

**Figure 4:**
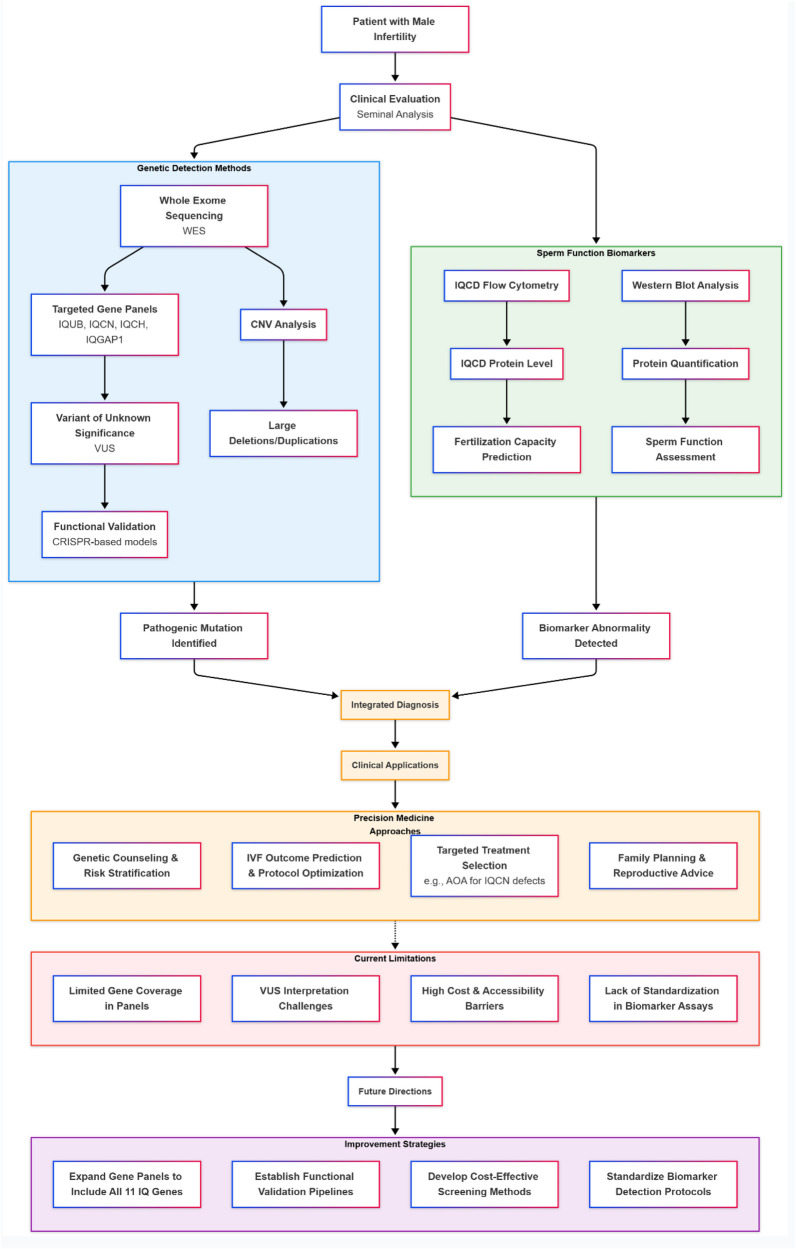
Diagnostic framework for male infertility leveraging IQ motif family genes.

This chapter outlines the diagnostic landscape for male infertility related to IQ motif family genes, integrating genetic detection with functional sperm assessment. As depicted in the accompanying diagnostic framework (Figure 4), the evaluation pathway begins with seminal analysis and proceeds through complementary approaches. Genetic detection methods, including whole-exome sequencing (WES) and copy number variation (CNV) analysis, have been pivotal in discovering pathogenic mutations in genes like *IQUB*, *IQCN*, and *IQCH* and establishing direct genotype-phenotype correlations (5, 16, 31). Targeted gene panels incorporating these actionable variants offer a cost-effective strategy for clinical screening (5, 16). In parallel, sperm function biomarkers are explored; notably, reduced levels of the acrosomal protein IQCD correlate with poor IVF outcomes, supporting its potential as a predictive biomarker (13). However, the clinical translation of these technologies faces significant hurdles, including limited gene coverage in panels, interpretation challenges for variants of unknown significance (VUS), high costs, and a lack of standardization for biomarker assays like IQCD quantification. The figure further illustrates how overcoming these current limitations—by expanding gene panels, establishing functional validation pipelines for VUS, standardizing assays, and developing cost-effective methods—is critical for advancing towards integrated diagnostics and personalized clinical applications, such as targeted treatment selection and improved ART counseling.

## 6 Treatment strategies for male infertility related to IQ motif family genes

This chapter details mechanism-informed strategies for managing infertility caused by defects in IQ motif family genes, spanning from clinically applied interventions to preclinical explorations and future concepts, as visualized in Figure 6.

### 6.1 Assisted reproductive technology (ART) optimized for specific genetic defects (clinically validated)

For infertility with a defined monogenic cause, ART procedures can be tailored to bypass the specific functional impairment.

#### 6.1.1 ICSI with artificial aocyte activation as a conditional intervention for IQCN-related infertility

Intracytoplasmic sperm injection combined with artificial oocyte activation (ICSI-AOA) represents a directly applicable intervention for specific genetic etiologies. In patients with homozygous loss-of-function *IQCN* mutations (e.g., nonsense or frameshift variants) who experience total fertilization failure with conventional ICSI, adjunctive AOA (e.g., with calcium ionophores) has enabled successful fertilization and live births in both mouse models and clinical cases (50), establishing it as a promising strategy to circumvent the sperm-specific oocyte activation defect. However, clinical outcomes are genotype-dependent. Other reported cases harboring different homozygous *IQCN* variants failed to achieve fertilization or pregnancy even with ICSI-AOA, ultimately requiring donor sperm (16, 34). Therefore, while ICSI-AOA is a validated option for certain *IQCN* defects, its efficacy is not universal and requires careful evaluation.

### 6.2 Exploration of gene and protein-based therapies (preclinical)

Preclinical studies explore strategies aimed at correcting the underlying cellular dysfunction, offering potential future avenues beyond ART.

#### 6.2.1 Targeting the IQGAP1 pathway for blood-testis barrier (BTB) dysfunction

The IQGAP1/CDC42 pathway is critical for maintaining BTB integrity. This demonstrates the feasibility of modulating this pathway, though direct *IQGAP1*-targeted therapy remains investigational, facing challenges in delivery vector optimization and long-term safety (6, 48).

#### 6.2.2 Protein delivery to restore critical interactions

Direct delivery of functionally deficient interacting proteins is another preclinical strategy. For instance, PIN1 stabilizes IQGAP1, and its deficiency leads to BTB failure and infertility in mice. In a proof-of-concept study, nanoparticle-mediated delivery of recombinant PIN1 protein to the testes of *Pin1*-knockout mice restored IQGAP1 function, BTB integrity, and partially rescued fertility (48, 49). While promising, such protein therapies face translational hurdles like short half-lives and the need for validation in higher species (49).

### 6.3 Future perspectives: rationale for germline gene correction

The establishment of clear genotype-phenotype correlations provides a rationale for considering future curative strategies. The causative link between biallelic *IQUB* loss-of-function mutations (e.g., c.942T > G) and asthenozoospermia is well-established (5, 14). *In principle*, genome editing tools like CRISPR-Cas9 could be applied to correct such mutations in spermatogonial stem cells, aiming to restore the production of functional sperm. However, this remains a prospective approach, with significant technical (e.g., off-target effects, delivery efficiency) and major ethical hurdles related to germline editing requiring resolution before any clinical application.

### 6.4 Summary

**Figure 5:**
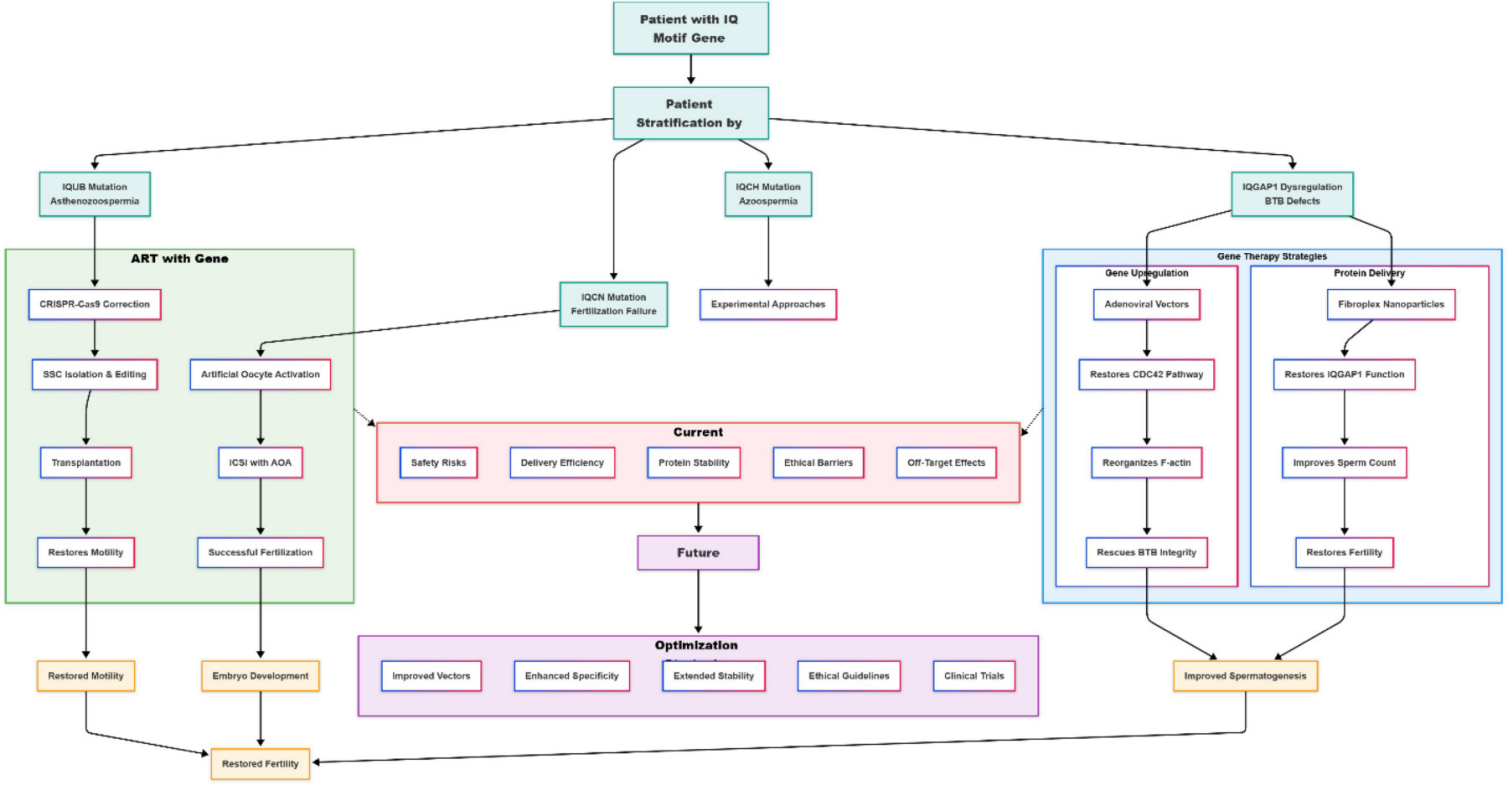
Overview of therapeutic strategies for male infertility associated with IQ motif family genes. The figure summarizes the transition from genetic diagnosis to mechanism-based management. Clinically applied ART (AOA-ICSI) effectively bypasses the fertilization failure in IQCN defects (16, 34). Preclinical strategies aim to correct underlying dysfunction: modulating the IQGAP1 pathway (48) or delivering therapeutic proteins like PIN1 (49) to restore BTB integrity. Future germline gene correction for mutations such as those in IQUB (5, 14) remains a prospective concept. This integrated approach exemplifies the progression toward precision medicine in male infertility.

This chapter outlines a spectrum of strategies for IQ motif gene-related male infertility: 1) Clinically validated ART optimizations, such as AOA-ICSI for certain *IQCN* defects (e.g., specific loss-of-function mutations) (16, 50), while noting that efficacy is not universal and may depend on mutation type (16); 2) Preclinical biological therapies targeting pathways (IQGAP1/CDC42) or delivering proteins (PIN1) to restore function (48, 49); and 3) Future conceptual frameworks for gene correction based on solid genetic etiology (5, 14). The translation of gene-based strategies faces substantial barriers. Collectively, these mechanism-informed approaches form an evolving framework for precision medicine, where diagnosis of a specific genetic defect can guide the selection of the most appropriate therapeutic pathway, as integrated in Figure 5.

## 7 Future prospects of research on IQ motif family genes and male infertility

### 7.1 Application of emerging technologies in gene research

Emerging technologies such as gene editing, single-cell sequencing, multi-omics, and computational tools offer transformative potential for elucidating the roles of IQ motif family genes in male infertility.

#### 7.1.1 CRISPR/Cas9 and single-cell sequencing: foundational tools for functional validation

CRISPR/Cas9 gene editing has been instrumental in establishing causal gene-phenotype relationships in animal models. For instance, its application validated the specific role of *IQUB* mutations in inducing sperm flagellar defects in mice (5). This technology provides a critical foundational platform for exploring future therapeutic concepts, such as gene correction in spermatogonial stem cells, though such applications remain firmly in the preclinical realm.

Single-cell RNA sequencing (scRNA-seq) has emerged as a powerful technique for delineating the cell-type- and stage-specific expression patterns of genes during spermatogenesis. Its application has begun to map the spatiotemporal expression of IQ motif family genes, providing crucial *in vivo* context for their proposed functions (31). For example, the enrichment of *IQCH* transcripts in spermatocytes aligns with its nuclear role in regulating alternative RNA splicing (7, 31), while the specific expression of *IQCD* in spermatids is consistent with its involvement in acrosomal formation and function (13). Applying scRNA-seq to human testicular samples from patients with diverse infertility etiologies represents a vital future direction to validate, refine, and expand these observations toward human pathophysiology.

#### 7.1.2 Computational tools: AI-based gene prioritization and network modeling

##### 7.1.2.1 AI-based gene prioritization for understudied IQ motif genes

Prioritizing understudied IQ motif genes [e.g., *IQCA1* ^(^51), *IQSEC3* 40] for functional validation is a challenge. Artificial intelligence (AI) and machine learning tools, trained on multi-omics datasets from reproductive tissues, hold promise for identifying high-priority candidates based on features like co-expression with known infertility genes or the presence of functional domains ^(^52, 53). *In the future*, such approaches could streamline the discovery of novel genotype-phenotype relationships.

##### 7.1.2.2 Network modeling to integrate fragmented pathways

IQ motif genes participate in interconnected pathways (Ca^2^⁺/CaM signaling, cytoskeletal dynamics, RNA splicing). Computational network modeling, including protein-protein interaction (PPI) analysis and systems biology models, provides a framework for integrating these pathways (54). *For example*, PPI databases can predict novel interactions (e.g., between IQCN and oocyte activation factors), which then require experimental validation (16). Similarly, *future* quantitative models could help simulate how perturbations in one pathway (e.g., IQGAP1-CDC42 signaling) might impact another (e.g., IQCH-mediated splicing), guiding hypothesis-driven research.

#### 7.1.3 Multi-omics integration with bioinformatics validation

Integrating genomics, transcriptomics, and proteomics (multi-omics) provides a comprehensive view of the molecular consequences of IQ motif gene dysfunction. Established bioinformatics workflows are essential for this integration. For instance, differential expression analysis of RNA-seq data from *Iqch*-KO mouse testes identified downstream genes and pathways (7), and tools like DESeq2 are standard for such analyses (55). Subsequent prioritization of candidate genes [e.g., *Tssk6* (47, 56)] and their functional validation [e.g., via CRISPR-KO (7, 47)] exemplifies a translational multi-omics pipeline. Applying this integrated approach to human samples is a critical next step.

### 7.2 Challenges and opportunities in IQ motif family gene research

Despite progress, several challenges persist in unraveling the role of IQ motif family genes in male infertility. For IQUB, the lack of commercial antibodies limits *in vivo* validation—functional studies rely solely on Western blotting with self-generated polyclonal antibodies, precluding immunofluorescence analysis of its localization in sperm flagellar radial spokes (5, 14). Similarly, IQCH research is hampered by species-specific functional differences: while IQCH is critical for spermatogenesis in mice, its role in humans remains unclear due to sequence divergence (78% amino acid identity between mouse and human IQCH, with only 65% conservation in the CaM-binding IQ domain) (7, 12).

Opportunities to address these gaps abound. High-resolution microscopy (e.g., cryo-EM) can elucidate the subcellular dynamics of IQ motif proteins during spermatogenesis—for example, visualizing IQUB's interaction with radial spoke components in sperm flagella. The integration of gene editing and multi-omics technologies promises to uncover novel genetic markers and therapeutic targets: combining CRISPR-mediated knockout of IQCN with RNA-seq, for instance, could identify downstream pathways regulating sperm-egg fusion. Large-scale population studies are also needed to address the gap in population-specific data—most IQ motif gene mutation reports focus on East Asian cohorts, leaving African, Latin American, and Indigenous populations understudied.

### 7.3 Importance of interdisciplinary cooperation in male infertility research

**Figure 6:**
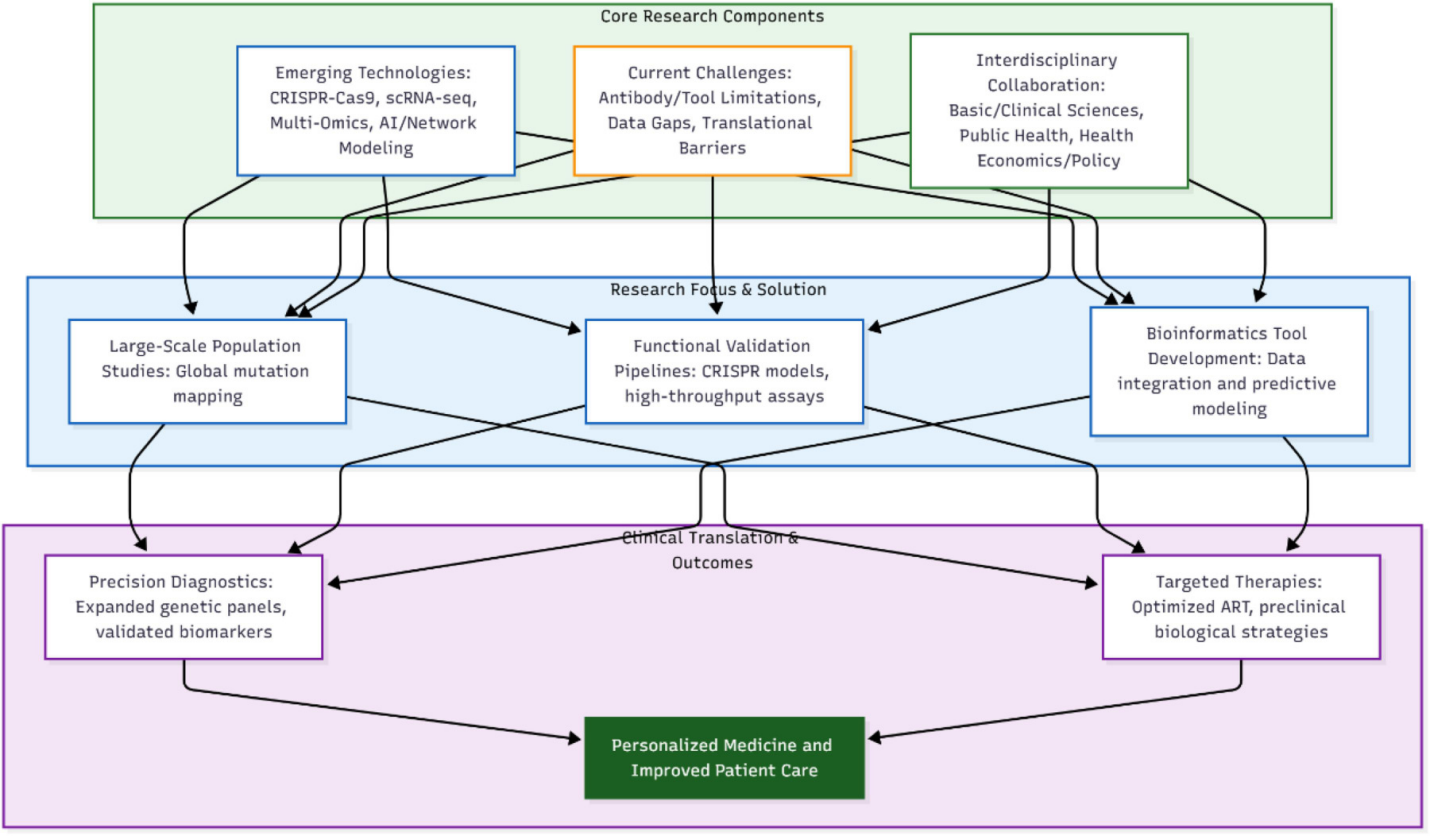
Future prospects of research on IQ motif family genes in male infertility.

Interdisciplinary collaboration is pivotal for advancing IQ motif gene research and male infertility care. As illustrated in Figure 6, this collaboration spans fields from endocrinology and genetics to biomechanics, psychology, public health, epidemiology, biostatistics, health economics, health policy, and health management.

From a public health perspective, large-scale screening programs for IQ motif gene mutations in high-risk groups (e.g., consanguineous families, individuals with a family history of infertility) could enable early intervention. Epidemiological studies can map the global prevalence of these mutations, while biostatistical models can identify associations between IQ motif gene variants and infertility phenotypes across diverse populations. Health economics research, meanwhile, can assess the cost-effectiveness of integrating IQ motif gene testing into clinical practice—for example, by reducing unnecessary assisted reproductive technology (ART) procedures in patients with IQCN mutations who require artificial oocyte activation (AOA), rather than conventional IVF.

Health policy and management play a crucial role in translating research into practice. Policies supporting standardized biobanking of testis tissue and genetic data can accelerate multi-omics research. Health management strategies, such as training clinicians in genetic counseling for IQ motif mutations, ensure equitable access to precision diagnostics. However, challenges remain, including ethical concerns over genetic data privacy (e.g., compliance with HIPAA, GDPR) and disparities in access to advanced diagnostics in low- and middle-income countries (LMICs)—issues that require policy solutions like subsidized screening programs and capacity building in LMICs.

### 7.4 Critical analysis and future directions for future prospects

While emerging technologies such as CRISPR-Cas9, single-cell sequencing (scRNA-seq), and artificial intelligence (AI) offer unprecedented tools to dissect the functions of IQ motif genes (5, 7, 31, 54), the field faces significant translational hurdles. Current research is constrained by technical and resource limitations, including the scarcity of high-quality antibodies for protein localization and the reliance on animal models that may not fully recapitulate human biology, as seen with IQCH (7, 12). Furthermore, a critical gap in population genetic data persists, with most pathogenic variants reported in East Asian cohorts, limiting the development of globally applicable diagnostics (5, 7, 16). To convert mechanistic insights into clinical utility, future efforts must be multi-faceted. First, advanced functional genomics—integrating multi-omics data from diverse human samples—is needed to map the complete regulatory networks and validate direct targets, especially for splicing regulator IQCH (7, 31). Second, overcoming the translational “valley of death” requires developing standardized research tools (e.g., antibodies, cell lines) and robust *in vivo* models to bridge species gaps. Finally, achieving true precision medicine necessitates sustained interdisciplinary collaboration that spans basic science, clinical genetics, epidemiology, and health policy (16). This integrated approach is essential to address data-sharing barriers, inform evidence-based guidelines, and ensure equitable access to diagnostics and therapies derived from IQ motif gene research.

### 7.5 Concluding paragraph: the integrative role of IQ motif genes in male reproduction and clinical care

IQ motif family genes serve as core integrators across the entire spectrum of male reproductive biology and clinical management, linking molecular function to infertility phenotypes and therapeutic strategies. During spermatogenesis, they orchestrate non-redundant processes: IQCH regulates alternative RNA splicing of testis-specific transcripts to ensure spermatid maturation, while IQGAP1 maintains BTB integrity via actin cytoskeleton reorganization—both critical for preventing spermatogenic arrest and azoospermia. In sperm function, IQUB modulates Ca^2^⁺/CaM-dependent inhibition of the p-ERK1/2/RSPH3 pathway to support flagellum radial spoke assembly, with mutations (e.g., c.842del, p.L281Pfs*28) causing asthenozoospermia despite normal sperm morphology; IQCN, by contrast, mediates acroplaxome stability and sperm head shaping, and its inactivation leads to acrosomal defects and fertilization failure due to impaired oocyte activation.

Clinically, these genes have transformed male infertility management: pathogenic variants such as IQUB c.942T > G and IQCN c.2388G > A have been incorporated into diagnostic recommendations for male infertility genetic testing, as evidenced in recent clinical studies and expert panels (16, 31, 35), to enable etiological diagnosis, and their molecular mechanisms guide targeted interventions—from AOA or transparent zone removal for IQCN-related fertilization failure to ICSI for IQUB-associated asthenozoospermia. As research advances, the integrative role of IQ motif genes will continue to bridge basic science and clinical practice, driving the shift toward personalized care for male infertility patients worldwide.

### 7.6 Summary

Chapter 7 outlines a comprehensive and translational research framework for studying IQ motif genes in male infertility. As visually synthesized in Figure 6, this model is built upon three synergistic pillars: emerging technologies, current challenges, and interdisciplinary collaboration.

Emerging technologies (e.g., CRISPR-Cas9, scRNA-seq, AI) provide the tools for functional discovery and systems-level analysis (5, 7, 31, 54). These advancements directly confront persistent current challenges, including technical limitations and population data gaps (5, 7, 12). To systematically address these obstacles, the framework necessitates interdisciplinary collaboration, integrating basic science with clinical research, public health, and policy (16).

The dynamic interplay of these core components, as depicted in Figure 6, channels into focused research streams—large-scale population studies, functional validation pipelines, and bioinformatics development—which in turn drive translational outcomes. These outcomes aim to deliver precision diagnostics, targeted therapies like optimized ART, and enhanced personalized care for patients.

In conclusion, Figure 6 serves as the unifying schematic for this chapter, encapsulating the entire translational pathway from molecular inquiry to clinical application. It effectively illustrates how the integration of innovation, challenge-driven research, and cross-disciplinary cooperation establishes a clear roadmap for advancing precision medicine in male infertility.
